# Are deep learning classification results obtained on CT scans fair and interpretable?

**DOI:** 10.1007/s13246-024-01419-8

**Published:** 2024-04-04

**Authors:** Mohamad M. A. Ashames, Ahmet Demir, Omer N. Gerek, Mehmet Fidan, M. Bilginer Gulmezoglu, Semih Ergin, Rifat Edizkan, Mehmet Koc, Atalay Barkana, Cuneyt Calisir

**Affiliations:** 1 Department of Electrical and Electronics Engineering, Eskisehir Osmangazi University, Eskisehir, Turkey; 2 Department of Electrical and Electronics Engineering, Eskisehir Technical University, Eskisehir, Turkey; 3 Vocational School of Transportation, Eskisehir Technical University, Eskisehir, Turkey; 4 Department of Computer Engineering, Eskisehir Technical University, Eskisehir, Turkey; 5 Department of Radiology, Manisa Celal Bayar University, Manisa, Turkey

**Keywords:** DNNs, Interpretability and reliability, Chest CT, Malignancy classification

## Abstract

Following the great success of various deep learning methods in image and object classification, the biomedical image processing society is also overwhelmed with their applications to various automatic diagnosis cases. Unfortunately, most of the deep learning-based classification attempts in the literature solely focus on the aim of extreme accuracy scores, without considering interpretability, or patient-wise separation of training and test data. For example, most lung nodule classification papers using deep learning randomly shuffle data and split it into training, validation, and test sets, causing certain images from the Computed Tomography (CT) scan of a person to be in the training set, while other images of the same person to be in the validation or testing image sets. This can result in reporting misleading accuracy rates and the learning of irrelevant features, ultimately reducing the real-life usability of these models. When the deep neural networks trained on the traditional, unfair data shuffling method are challenged with new patient images, it is observed that the trained models perform poorly. In contrast, deep neural networks trained with strict patient-level separation maintain their accuracy rates even when new patient images are tested. Heat map visualizations of the activations of the deep neural networks trained with strict patient-level separation indicate a higher degree of focus on the relevant nodules. We argue that the research question posed in the title has a positive answer only if the deep neural networks are trained with images of patients that are strictly isolated from the validation and testing patient sets.

## Introduction

The society of biomedical image processing has an abundance of image and object classification publications due to the great success of various deep learning methods. The biomedical images in various automatic diagnostic cases may consist of stand-alone images (such as X-rays) or batch scans (such as Computed Tomography (CT), Magnetic Resonance Imaging (MRI), etc.) In batch processes, scans from different offsets are obtained to observe the tissues of a person. Deep learning has been shown as a prevalent and effective algorithm in the diagnosis of many medical images [35]. However, it has also been criticized for not being reliable because of its lack of complete explainability [16, 25]. The method may provide good diagnosis scores, yet it may be difficult to understand the underlying reason for a possible success. Besides, the reported classification accuracy of some publications cannot be reproduced in consecutive experiments.

Separating data into exclusive train/test sets is required to evaluate the performance of a supervised machine learning (ML) algorithm. The splitting is typically performed to contain fractions of data for these sets in a random manner. In a related study, Goodfellow et al. asserted that the training and testing data could be split with a probability distribution that obeys the independent and identically distributed (iid) assumption [10]. Unfortunately, many ML-based diagnosis attempts in the literature did not handle image datasets obtained from batch scans with sufficient care regarding the *independence* condition as explained above. In most cases, the test-train separation of images from multiple scans was done randomly, providing images from the same scan to appear in the training and the test or validation sets. Since such a situation is a direct violation of the independence requirements, we investigate the effect of such *unfair* train-test splitting on the performance of ML methods in terms of detection accuracy and overall algorithmic interpretability. Besides, the efficiency and interpretability improvement under the strict (i.e., patient-wise) separation of train and test (or validation) data splitting case is studied in this work.

As a case of the careful test-train separation problem, we consider lung nodule malignancy detection in CT scans, where the literature contains several deep-learning algorithm results.

Two survey papers reviewed available Computer-Aided Design (CAD) systems applying deep learning to CT scan data for lung nodule detection, segmentation, classification, and retrieval [12, 41]. They argue the advance of deep learning, define various important characteristics of lung nodule CAD systems, and evaluate the performance of certain studies against different databases such as LIDC, LIDC-IDRI, LUNA16, DSB2017, NLST, TianChi, and ELCAP. In the selected classification studies, the accuracy rates range from 71% to 99.6%. High accuracy results arise from the inclusion of different CT images belonging to the same patient in both training and test sets. Throughout this paper, we call this kind of test/train splitting the UNFAIR case. In this case, only image-wise cross-fold validation technique is used [1, 15, 20, 21, 24, 26, 33, 35, 42–45, 49]. In these studies, LIDC-IDRI database is widely used with various ML-based classificatio methods such as convolutional neural networks (CNN) [32, 35], an interpretable and multi-task learning CNN [42], an algorithm enhancing the optimization function in CNN [22], pre-trained ResNet-50 models [43], multi-view knowledge-based collaborative (MV-KBC) deep model [44], recurrent neural networks (RNN) with softmax [1], forward and backward generative-adversarial networks (F &BGAN) with multi-scale VGG16 (M-VGG16) network [49], texture - shape - deep model learned information fusion (Fuse-TSD) [45], multi-crop CNNs (MC-CNN) [33], lightweight and multiple view sampling based Multi-section CNN architecture [26], end-to-end deep multi-view CNN [15], k-Nearest Neighbor (k-NN) and Multi-Layer Perceptron (MLP) [24], and multi-view CNN (MV-CNN) [20]. Using the compatible datasets, the accuracy rates of these studies were reported to vary between 84.15% and 98.31%.

Other DL methods that report very high classification accuracies include the work by Nibali et al, where the effect of curriculum learning, transfer learning, and network depths on malignancy classification with ResNet was investigated, and an accuracy rate of 89.9% was achieved for the LIDC-IDRI database [21]. In a work by Shaffie, et al, the LIDC database was used with a denoising autoencoder (DAE) and 3D Resolved Ambiguity Local Binary Pattern (3D-RALBP) methods, and an accuracy rate of 94.95% was obtained [30]. In another work, a DNN optimization was performed via Modified Gravitational Search Algorithm (MGSA) for CT images, and the resulting network (named Optimal Deep Neural Network - ODNN) was reported to achieve 94.56% classification accuracy for lung cancer in ELCAP database [18]. When the CT images taken from the Cancer Imaging Archive were used for lung nodule classification, extreme accuracy rates of 99.51% and 97.14% were obtained using k-NN with AlexNet & mRMR feature extractor in [38], and LDA classifier in [2], respectively. In another study, Tran et al. suggested a new 2D architecture for a deep CNN using focal loss, and obtained an accuracy rate of 97.2% for the LUNA16 database [39]. In a final example, Shafi et al use the LUNA16 database with capsule NN-based SVMs to achieve a classification accuracy of 94% for cancerous lung nodules [31].

The train/test splitting approach of the above-mentioned methods all share a common mechanism of using random image assignments to the test and train sets without considering whether a patient’s images appear in both sets or not. All of these manuscripts report that the whole image lot was shuffled completely, and train-test separation was done randomly. In such a case, some images from the same patient scan may go to the training set, while the rest may go to the test set, making an unfair splitting that is prone to overfitting with too high accuracy results. Therefore, these extreme accuracy rates *could* be attributed to a possible overfit due to the above-mentioned unintentional leak of same-batch image data to both training and test sets.

Contrary to such unfair train/test splitting, a fair splitting approach is also possible, where one carefully assigns distinct patients’ scan images to train and test sets. The literature also contains several papers, where this attention was paid [11, 17, 19, 23, 40]. In these studies, the maximum accuracy rates of around 75% - 90% were obtained with various DL techniques. For instance, Kumar et al use deep features extracted from an autoencoder along with a binary decision tree [17] for a part of LIDC database, Liao et al use a 3-D version of the RPN using a modified U-net and a leaky noisy-OR model for DSB2017 database [19], Gruetzemacher and Gupta use a CNN model for LIDC-IDRI database [11], Paul et al use VGG-s CNN models for NLST database [23], and Utkin et al use an ensemble of triplet neural networks for LUNA 16 database [40]. In [13] and [37], authors created their own databases, and the accuracy rates of 75.2% and 71.1% were obtained using artificial intelligence (AI) systems obtained from the union of various CNNs.

It is clearly visible from the second group of works that the reported classification accuracies are lower than those reported in the first group, where unfair data splitting was performed. Although overfitting due to unfair test-train dataset splitting seemingly gives higher accuracy results, the reliability of the results could be questionable from the following aspects:

- Do these trained ML techniques still provide high accuracy for a completely new *challenge* data set?
- Do these trained ML techniques perform classifications by really focusing on the actual nodule positions (marked by radiologist experts)?
- Hence, are these techniques *interpretable*?

A follow-up question automatically arises:

- If we perform strictly fair test-train splitting, does this improve performance on the challenge data set and interpretability?

A similar concern of strict patient-wise training/validation/test separation was mentioned in a comprehensive review by Loizidou et al for a different problem of mammography classification. They propose that images and image labels (i.e., ROIs) of the same patient should be incorporated into the training, validation, or test mammography datasets. They also express concern regarding the high classification accuracy rates reported in various papers that failed to perform this separation, as they render the performances unverifiable for new patient cases in real-life.

In this study, we explore this idea in the context of CT scans, demonstrating the invalidity of unfair training accuracy results numerically. Furthermore, we show that deep neural networks trained using unfair random image splitting are incapable of focusing attention on indicator regions of CT images (i.e., nodule regions), which renders the results completely non-interpretable. This study provides experiments comparing the reliability of deep learning algorithms for lung nodule classification by implementing fair and unfair data splitting.

## Materials and method

**Fig. 1:**
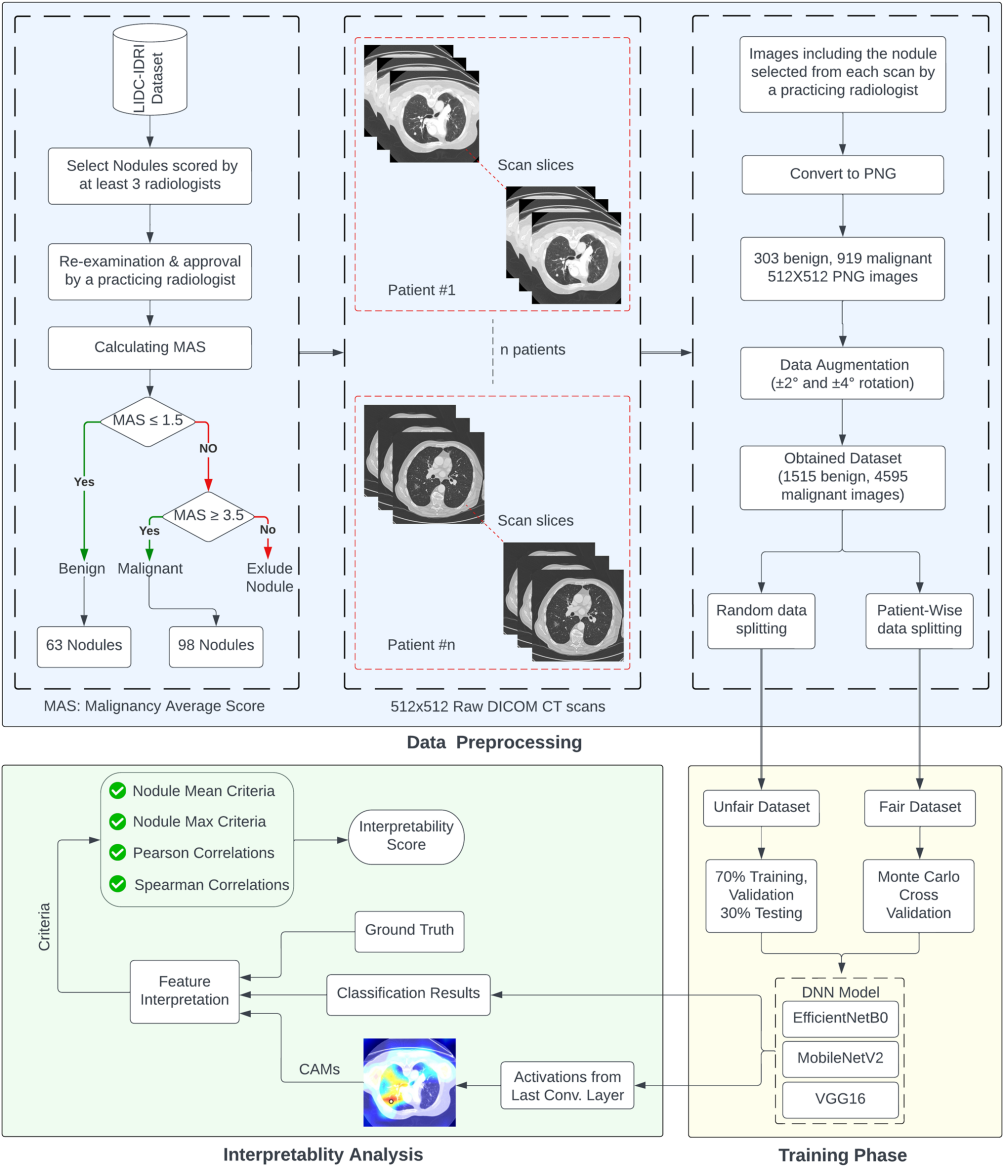
Block diagram of the followed data preprocessing, training, and interpretability analysis approach

Starting from the construction of the dataset and ending at the interpretability measurements, the overall methodology comprises several process steps. The complete layout of the methodology is shown as a large flowchart in Fig. 1. The detailed steps of the process are provided in the following subsections.

### Dataset

**Fig. 2:**
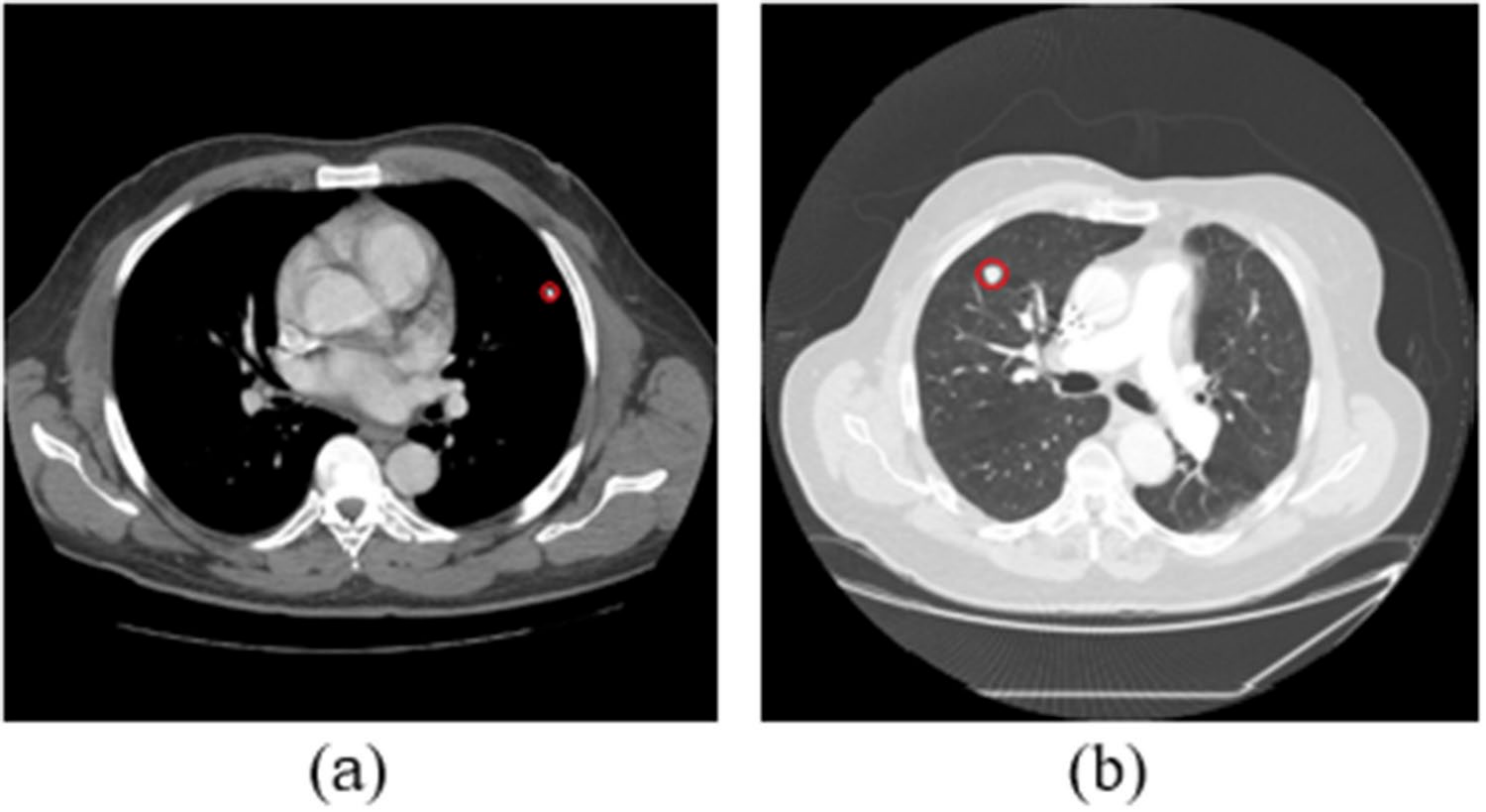
Axial CT images of **a** Benign, **b** Malignant pulmonary nodules. Nodules are circled with red color on the images

Our study utilized a subset of the LIDC-IDRI dataset, a large thoracic CT scan collection from 1010 patients, publicly available and initially created by NCI in 2001, with later expansions [5]. This dataset, one of the largest of its kind, encompasses 1018 CT scans acquired using various scanner devices and parameters. Each scan is accompanied by an XML file detailing blinded and unblinded nodule diagnoses and reports from four radiologists. The radiologists categorized nodules based on diameter and provided final decisions and malignancy scores for nodules $\ge$ 3 mm. Individual interpretations were maintained without consensus. We extracted our study’s dataset based on specific criteria from the ‘LIDC Nodule Size List’ document. This subset included only nodules assessed by at least three radiologists, further validated by a practicing radiologist. Nodules were classified as benign (average score $\le$ 1.5) or malignant (average score $\ge$ 3.5), resulting in 63 benign and 98 malignant nodules. This classification threshold was chosen to ensure high diagnostic certainty, as nodules with scores $\le$ 1.5 reliably exhibit benign characteristics, while scores$\ge$ 3.5 are strongly indicative of malignancy. This approach maintains the accuracy and consistency of our findings, ensuring that our analysis is based on data with clear diagnostic indicators. Using ‘noduleID’ and ‘imageZposition’ parameters, 303 benign and 919 malignant nodule images were acquired from $512 \times 512$ DICOM format and converted to PNG using MicroDicom software. An example of both benign and malignant CT images is shown in Fig. 2.

To mitigate overfitting, we augmented the data by rotating each image by ±2 and ±4, increasing the dataset to 1515 benign and 4595 malignant images. We then split this augmented dataset into training and test sets using two methods: ‘unfair’ (image-wise-random) and ‘fair’ (patient-wise-random). The unfair method resulted in training, validation, and test sets with 969/2940, 410/1241, and 136/414 benign/malignant images, respectively. In contrast, the fair splitting method ensured no patient’s CT scans were used across multiple phases.

### Deep neural network architectures

DNNs, particularly their convolutional implementations, have become the gold standard in today’s classification problems. In this paper, the classification task is realized by using three well-known DNN architectures, explained herein.

The first adopted DNN is the popular VGG-16 architecture, which was proposed by Simonyan and Zisserman at Oxford University in 2014 [34]. VGG-16 contains 16 groups of layers, taking RGB images with a resolution of 224 × 224 pixels as input. It has a convolution kernel with the size of 3 × 3 and a maximum pooling layer with the size of 2 × 2. It is one of the most widely used architectures in various pattern recognition studies despite its relatively slow training process.

The next used DNN is EfficientNet, which is flexible in scaling and balancing certain parameters such as depth, width, and resolution with the help of a compound coefficient [36]. It aims to lower the calculation cost by dividing the conventional convolution into two phases. Along with that, it diminishes possible losses resulting from the use of Rectified Linear Units (ReLU) by utilizing a linear activation function at its final layer. Various variants of this architecture exist in the literature, and we have chosen EfficientNetB0 as an example architecture in our experiments.

The third utilized network, MobileNet, is a newly developed NN architecture by Google researchers. It is adapted mainly to mobile devices due to its small memory and computational requirements and resultant low-latency properties [27]. MobileNetV2 is the second version of MobileNet that adds bottleneck layers and alters the filtering operations for improved performance. Consequently, our experiments incorporated this version as a use case.

### Training procedure

Google Colaboratory (i.e., Colab) was used as an environment for the experiments. The environment provides a tool for writing and executing Python code, especially applicable for machine learning tasks [6]. Keras with Tensorflow backend was used to import the DNN architectures. End-to-end binary classification was carried out through the above-mentioned three architectures, all pre-trained using ImageNet. The networks were fine-tuned with a final binary softmax layer to achieve a binary classification. The images were fed to the networks after resizing to the network’s corresponding default image size.

Training parameters used in the experiments are given in Table 1. The starting value of the learning rate is reduced to one-tenth if no validation accuracy improvement is seen for several epochs.

**Fig. 3:**
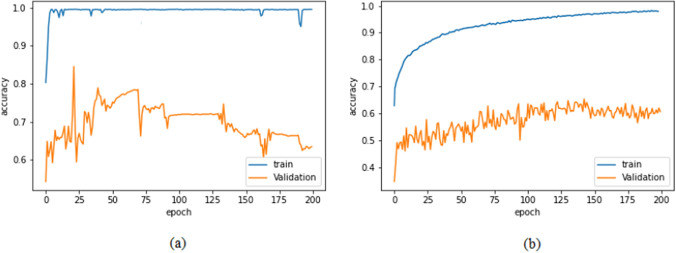
Training and validation accuracies for both **a** unfair and **b** fair train-validation procedures using EfficientNet. **a** indicates how unfair data splitting can lead to high training accuracies with a lack of convergence in the validation set, which effectively demonstrates the issue of overfitting

In the unfair train-validation process, 70% of the dataset is utilized for training and validation, while the remaining 30% is set aside for testing. This method has a chance to feed different images from the same CT scan into training, validation, and testing. This may cause the network to learn the patient instead of the malignancy markers. Since the test set also contains images from these patients, the possible patient-learning process could yield high, but unjustified accuracy results, which we call "unfair". Such overfitting at early stages is demonstrated in Fig. 3a.

**Fig. 4:**
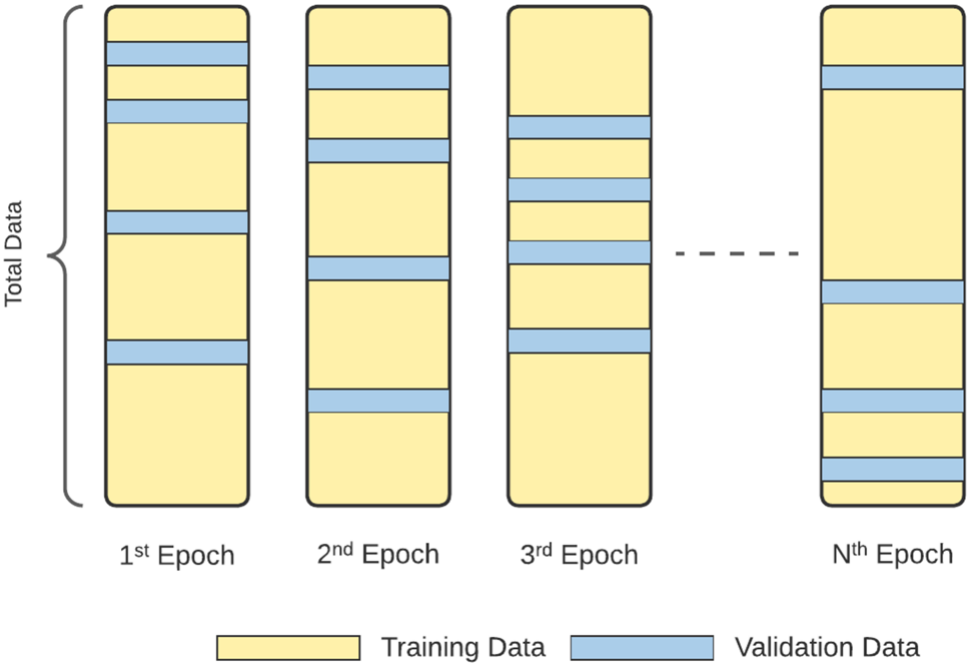
Monte Carlo Cross Validation. The diagram shows that patients of the validation set change each time a new epoch starts

To avoid overfitting and achieve reliable accuracy results, separate folders that contain CT scans of different patients were constructed in the second experimental set, which we call the FAIR training procedure. Monte Carlo Cross Validation (MCCV) [46] was applied to shuffle the patients (with all of their CT scan images) during training, validation, and testing, as illustrated in Fig. 4.

In developing our Monte Carlo Cross Validation (MCCV) approach for patient-wise data splitting, we aimed to emulate the decision-making process of practicing radiologists, who assess all relevant slices within a single scan and focus exclusively on data pertaining to that specific patient before making a diagnosis. This patient-specific approach ensures both a comprehensive and individualized analysis, mirroring the radiologist’s method of thorough, patient-wise evaluation. In a broader context, the concept of mimicking real-world behaviors or phenomena for optimization is not new and has been explored in various fields. Studies such as [3, 4, 8, 9, 14, 48], have demonstrated innovative approaches where natural behaviors are modeled to develop sophisticated optimization techniques. These methodologies highlight the potential of drawing inspiration from nature and real-world scenarios to enhance the efficacy of algorithms in diverse engineering applications. Our medical field application, while distinct, aligns with this broader theme of simulating real-world processes for optimization. The MCCV method in our FAIR training procedure is a testament to the effectiveness of such inspired approaches. By shuffling patient data sets, we aim to avoid overfitting and ensure a more reliable and robust assessment of CT scans, akin to the careful and patient-specific analysis conducted by radiologists. This methodology not only improves the accuracy of our deep learning models but also reinforces the principle of fair and unbiased data analysis in medical diagnostics.

The improvement in the learning process and validity of the reported accuracy results are analyzed. Figure 3b clearly shows that the proposed training process gradually improves in time, and no inconsistent overfitting occurs. Furthermore, the resultant networks provide more reliable accuracy results, as will be explained in Sec. 3.

**Table 1:** DNN training parameter settings

Parameter	Value
Learning rate	0.0001
Epoch number	50–200
Batch size	32
Optimizer	ADAM

### Interpretability analysis

In the context of Deep Neural Networks (DNNs), which frequently function as enigmatic ’black boxes’ with limited interpretability, the visualization of the decision-making process becomes essential for evaluating the model’s reliability. Class Activation Mapping (CAM) is a visualization tool that can be employed to assess the post-hoc interpretability of the network. It is intricately designed to identify and highlight the discriminative regions within an image that are crucial for a DNN’s decision-making process [7]. CAM accomplishes this by creating color-coded representations, often referred to as heat maps, which visually articulate the magnitude of a phenomenon, thereby shedding light on the critical aspects influencing model decisions [29, 47].

The creation of heat maps involves calculation of gradients using both the output of the last convolutional layer and the output of the deep model. Neuron weights are then acquired via the average pooling of these gradients. The values in each layer of the last convolutional block are multiplied by their corresponding neuron weights, and the average and maximum of these values are computed to generate the heat map. The heat map is then normalized and subjected to color mapping before being combined with the original image. By highlighting the areas of an image that are most influential in the model’s prediction, heat maps can provide insight into the internal workings of DNNs and their ability to perform complex tasks.

In order to quantify the interpretability, this study employs two prominent scoring methodologies over the calculated heat maps. The first scoring method focuses on the locality of the attention heat map values that match to the tumor location. The location matching can be measured by either averaging activation intensities inside the nodule regions or by picking the highest value inside the actual tumor region.

The second method measures the structural similarity of the nodule regions and compares them to the structure of the shape obtained from the heat map image. It is argued that if the heat map shape structurally matches the nodule shape, it indicates a high interpretability score. Two well-known correlation techniques measure the pixel layout similarity between two images: the Pearson and the Spearman correlation [28]. Pearson correlation evaluates the linear relationship between two images, whereas Spearman correlation is a more general measure that evaluates the monotonic relationship between two images. These classical correlation values are evaluated to find the shape-wise relation between the focus heat map values and binary morphological shape corresponding to the ground truth nodule pixels. It is argued that a high correlation (closer to one) would indicate that the heat map shows a correct focus on the nodule regions, whereas smaller correlation values would mean an incorrect, hence an uninterpretable focus.

## Results

The described methodology was applied to the CT scan images to see the effect of fair and unfair train/validation/test separation in the classification and interpretability perspectives.

### Classification results

The aforementioned three DNN architectures; MobileNetV2, EfficientNetB0, and VGG16, were trained and validated, first through the unfair training-validation separation, and then through fair dataset splitting by MCCV. Table 2 compares the classification accuracies for the unfair and fair experiments of each architecture. As expected, the architectures tend to report misleadingly high accuracies when they are unfairly trained and tested, while they reach lower (but correct) accuracy values when patient-wise data splitting is carried out, and different CT scans are used for testing.

**Table 2:** Classification accuracies obtained by implementing fair and unfair training–testing for both test and challenge datasets

DNN architecture	Number of epochs	Fair		Unfair	
		Test Acc	Challenge Acc	Test Acc	Challenge Acc
MobileNetV2	50	0.7365	0.7151	0.9836	0.7402
	200	0.7081	0.6702	0.9855	0.6693
EfficientNetB0	50	0.7035	0.6812	0.9873	0.4331
	200	0.7194	0.7188	0.9873	0.4252
VGG16	50	0.6881	0.6432	0.9909	0.3701
	200	0.7220	0.6933	0.9873	0.3701

In order to assess the correctness and validity of the reported test accuracies, CT images of a completely isolated set of patients (called the challenge set) were applied to the trained networks. The challenge dataset, intentionally separate from the train-validation-test groups used in both fair and unfair training scenarios, comprises images from two distinct groups: 93 images of malignant nodules from 9 patients, and 34 images of benign nodules from 7 patients. These patients were exclusively selected for the challenge set and were not part of the train-validation-test process. A practicing radiologist selected these patients to ensure a diverse and comprehensive representation of nodule characteristics, with a focus on the variation in lung anatomy at different stages of the scan. This strategic selection was pivotal in robustly testing our model across varied scenarios, including shifts in lung shape and nodule appearance across different scan sections.

The obvious observation is that the reported test accuracies (left-side column) of the unfairly trained network are far from being valid for the challenge set (right-side column), whereas the challenge performance of the fair-trained network is totally consistent with the reported test accuracies. Interestingly, certain networks (i.e., EfficientNet and VGG16) result in an extreme failure in the challenge dataset when they are unfairly trained, giving an impression that overfitting and patient-learning could be a more pronounced issue in these networks.

**Fig. 5:**
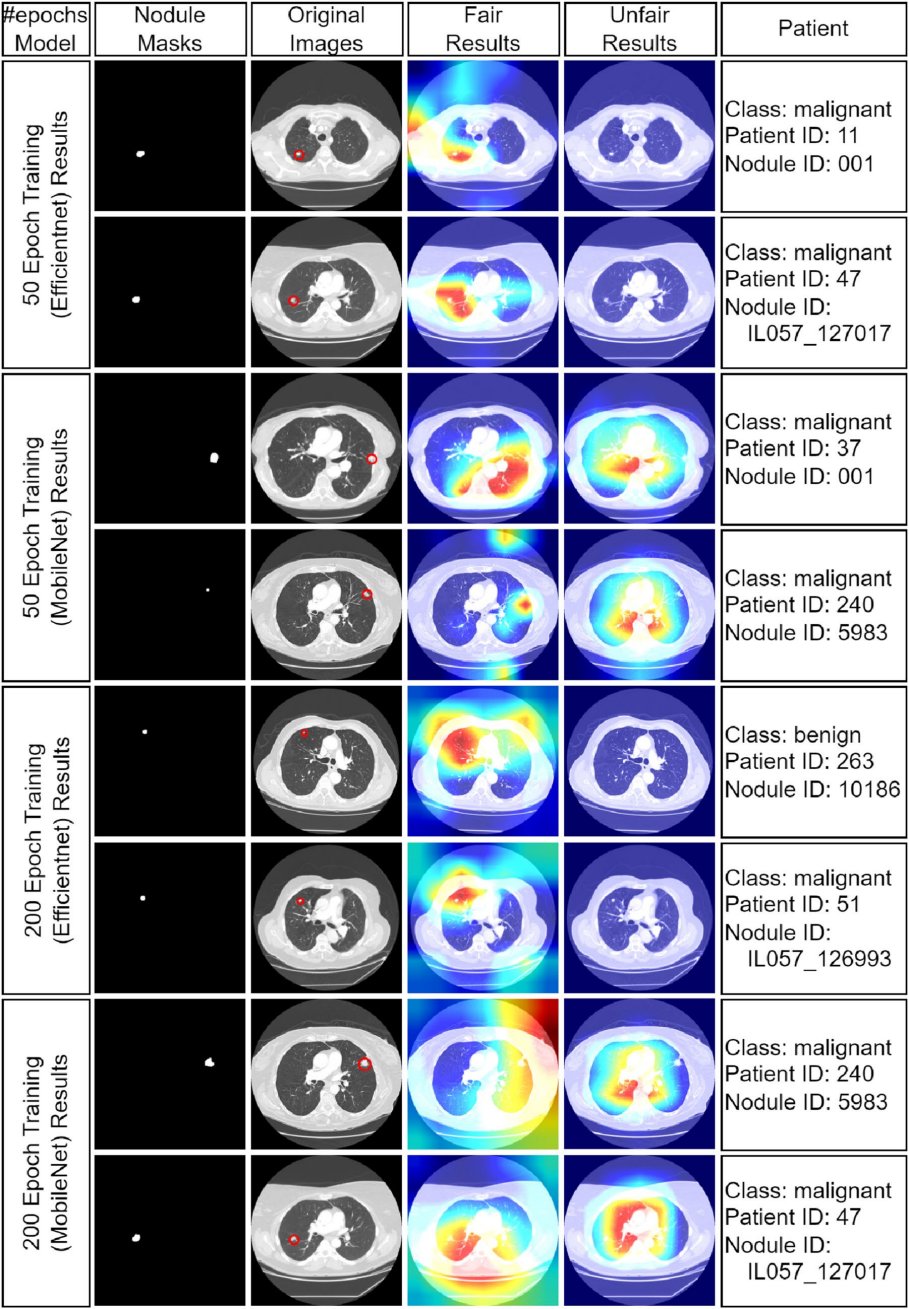
Heat map visualizations (CAMs) for eight randomly selected test images. The first column indicates masks of the nodules, the second column indicates original CT images, the third column indicates fair model CAMs, and the fourth column indicates unfair model CAMs. Nodules are pointed with the red circles in the original CT images

### Interpretability results

**Fig. 6:**
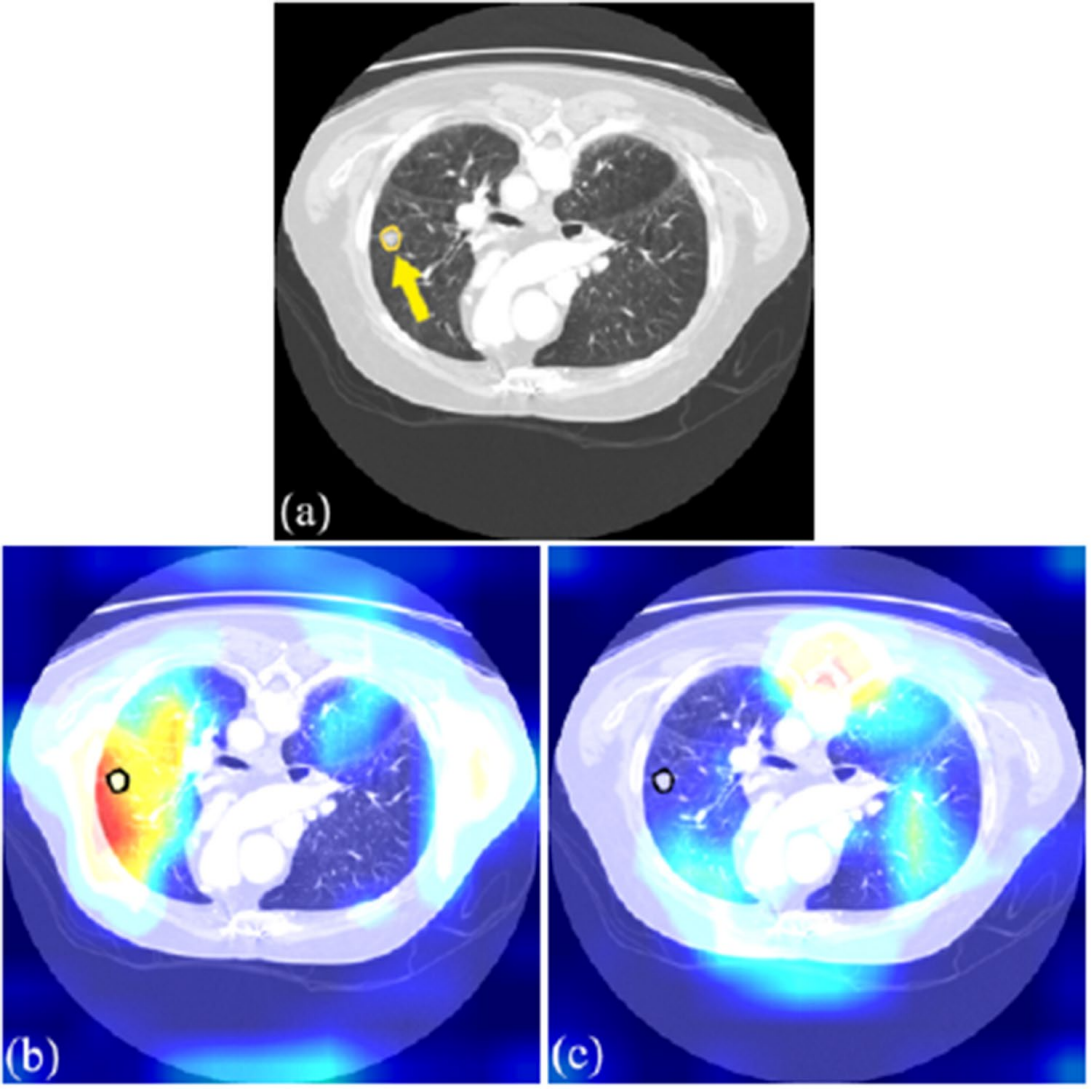
**a** Example of a CT image with a malignant nodule from LIDC-IDRI (with a yellow arrow showing nodule place); **b** corresponding fair model heat map output; **c** corresponding unfair model heat map output

**Table 3:** Heat map values of several patients for Unfair and Fair cases concerning nodule max and nodule mean criteria

Patient IDs	Unfair		Fair	
	Nodule max	Nodule mean	Nodule max	Nodule mean
11	0	0	0.8882	0.7908
37	0.3824	0.2997	0.8030	0.7076
47a	0	0	0.8677	0.8116
47b	0.6942	0.5901	0.7444	0.7046
240a	0.3762	0.1990	0.6319	0.4167
240b	0.6607	0.5152	0.7529	0.6972

**Table 4:** Pearson and Spearman correlations of heat map and ground truth nodule shapes for Unfair and Fair cases

Patient IDs	Unfair		Fair	
	Pearson Corr	Spearman Corr	Pearson Corr	Spearman Corr
11	0.0133	0.0286	0.0624	0.0548
37	0.0306	0.0375	0.0764	0.0668
47a	0.0112	0.0183	0.0563	0.0561
47b	0.0473	0.0462	0.0447	0.0465
240a	0.0053	0.0132	0.0327	0.0353
240b	0.0226	0.0300	0.0671	0.0695

To demonstrate interpretability, 8 images were randomly chosen from the test set for interpretability analysis with both fair and unfair models. Heat maps, generated from the last convolutional layers of MobileNetV2 and EfficientNet, highlighted lung regions influencing model decisions (Fig. 5). Red areas indicate stronger activations. The unfair model often incorrectly focused on non-tumor areas for malignancy predictions, while the fair model correctly concentrated on tumor regions. This indicates the unfair model’s unreliability while achieving misleadingly high accuracy results, likely stemming from overfitting due to improper train/test splitting.

Figure 6a displays a malignant CT image (ID 54) from the LIDC-IDRI dataset with the tumor region marked. Both fair and unfair models correctly classify its malignancy. However, differences in model reliability are revealed by overlaying their activation heat maps on the CT image (Figs. 6b and c). The fair model’s heat map shows high activation around the tumor, indicating accurate focus, whereas the unfair model’s heat map lacks focus on the tumor area, suggesting unreliable reasoning, possibly due to overfitting from improper train/test splitting. This underscores that the unfair model’s correct classification may be coincidental.

Using the images in Fig. 5, the average and maximum heat map values inside the nodule regions using the unfair models are provided in Table 3. Both the maximum and the average heat maps inside the nodule regions using the unfair models are significantly lower than the values obtained using the fair models.

Regarding the shape matching scores between heat map images and actual nodules, Table 4 shows Pearson and Spearman correlation values for the set that was used in Fig. 5. In this table, the stronger correlations between the nodule regions and the provided heat maps for the fair models indicate that the fair model focuses better to the actual nodule regions and causes a more reliable machine learning process as compared to the unfair model, where these correlation values are visibly lower. The results in Tables 3 and 4 focus only on the instances from Fig. 5 to provide a concise illustration of the potential pitfalls in data handling and model interpretation, rather than an exhaustive analysis of the full test set, which comprises 558 images from 28 patients.

## Discussion

Following the extensive set of experiments, our experimental observations show that patient-level separation is crucial in the training and testing of deep neural networks for reliable and interpretable lung nodule classification in CT images. Experimental results reveal a critical discrepancy between the perceived and actual performance of DNNs depending on the data-splitting methodology employed. Despite seemingly high reported values for classification accuracy, careless image splitting without patient-wise separation in training and testing can lead to unreliable and unfair results that cannot be verified in new challenge datasets. This discrepancy between test and challenge sets highlights the risk of overfitting in models that are trained without patient-level separation, where the model may inadvertently learn patient-specific features rather than generalizable markers of malignancy. On the other hand, patient-wise splitting in the training and testing process provides consistent, correct, and reliable results for accuracy percentages. This consistency is crucial in a clinical setting, where the ability to generalize to new patient data is a necessity.

Models trained with patient-wise splitting not only perform better in terms of consistent accuracy but also demonstrate a heightened focus on relevant nodule regions. This finding is crucial for clinical applications, as it ensures that the model’s decision-making process aligns with the critical features identified by medical professionals. The dual approach in interpretability analysis - both qualitative (heat maps) and quantitative (correlation analysis) - provides a comprehensive understanding of how the model processes the images and what features it focuses on.

## Conclusion

Lung cancer is among the top causes of cancer deaths globally, highlighting the need for early and precise distinction between benign and malignant lung nodules to improve health care. The rapid advancement of machine learning (ML) and deep learning (DL) for automated lung nodule classification in CT scans has been instrumental, as evidenced by the vast number of related publications. While deep neural network (DNN) methods are particularly promising in this domain, there’s a tendency in research to prioritize reported accuracy over the true reliability of these diagnostic systems.

Contrary to the desire to achieve the highest possible classification accuracy results by the engineering community, practicing radiologists always prefer a reliable and interpretable method, as incorrect reasoning of the ML method may pose catastrophic results in real-life health cases. With a collaboration of engineers and radiologists, the presented teamwork proposes an interpretability scoring methodology and concludes a necessity of strict patient-wise splitting of train/validation/test datasets in order for the presented accuracy rates to be reliable and the learning system to be interpretable. Based on our findings, we recommend the following best practices for deep neural network training and testing for lung nodule classification in CT images:

- Strictly separate the training, validation, and test datasets at the patient level to ensure reliable and interpretable results.
- Verify the interpretability of the trained networks by analyzing attention heat map values and correlation analysis between heat map images and the nodule regions.
- Report accuracy percentages for both overall performance and performance on new patient images to ensure the generalization of the deep neural network to new patients.
- Provide clear documentation of the dataset splitting methodology in reports related to DNN training and testing for lung nodule classification in CT images.

The provided observations indicate that further care must be taken in ML and DNN applications of crucial medical applications such as benign/malignant classifications or diagnosis aids for achieving better reliability and real usability in medicine.

## Data Availability

Not applicble.
